# Enhancing Polyphenol Delivery and Efficacy Using Functionalized Gold Nanoparticles: Antioxidant and Antibacterial Properties

**DOI:** 10.1155/bca/3836765

**Published:** 2025-06-09

**Authors:** Siham Bouaouz, Miriam Chavez, Cornelia E. Peña González, Daniel Rojas, Alberto Escarpa, Paula Ortega, F. Javier de la Mata

**Affiliations:** ^1^Department of Organic and Inorganic Chemistry, Research Institute in Chemistry “Andrés M. del Río” (IQAR), University of Alcala, Madrid, Spain; ^2^Department of Analytical Chemistry, Physical Chemistry and Chemical Engineering, Institute in Chemistry “Andrés M. del Río” (IQAR) University of Alcala, Madrid, Spain; ^3^UCAM-SENS, Universidad Católica San Antonio de Murcia, UCAM HiTech, Avda.Andres Hernandez Ros 1, 30107, Murcia, Spain; ^4^Networking Research Center on Bioengineering, Biomaterials and Nanomedicine (CIBER-BBN), Spain, Institute “Ramón y Cajal” for Health Research (IRYCIS), Madrid, Spain

**Keywords:** antibacterial, antioxidant, dendron, gold nanoparticles

## Abstract

Research into the use of nanoparticles to enhance the delivery and efficacy of polyphenols is a topic of growing interest in the fields of nanotechnology, pharmacology and food science. Nanoparticles, due to their small size and high surface area, can improve the stability, solubility and bioavailability of polyphenols. Combining polyphenols with other bioactive compounds within nanoparticles can create synergistic effects, enhancing their overall therapeutic potential. In this work, we present a new polyethylene glycol (PEG) capping ligand modified with caffeic acid (CA), HS-PEG-CA and two types of gold nanoparticles: (i) coated with a PEG-thiol derivative functionalized with CA (HS-PEG-CA) (homofuncionalized NP) and (ii) HS-PEG-CA cationic carbosilane dendrons with antibacterial properties (heterofuncionalized NP). The antioxidant capacity of the CA, in three systems, has been studied by different techniques such as FRAP, DDPH and cyclic voltammetry, demonstrating that it is preserved when it is supported on the NP and increases when it is part of the PEG ligand. In addition, heterofuntionalized NP showed activity against *S. aureus* and HS-PEG2K-CA ligand can effectively anchor to gold substrates.

## 1. Introduction

The use of nanotechnology in the field of medicine has opened the door to the search for nanomaterials that act as new therapeutic agents as an alternative to traditional treatments. From all the nanosystems described in the literature, metal nanoparticles (MNPs) offer several advantages such as a high surface area-to-volume ratio, which allows the introduction of a large number of ligands, high chemical and thermal stability, permeability, and good biocompatibilities [1, 2]. Recently, there has been a growing interest in the development of nanoparticles with antioxidant properties, since oxidative stress has been related to the progress of numerous pathophysiological diseases [3]. In this process, an unbalance of prooxidant/antioxidant species in favour of prooxidants is produced, increasing the levels of free radicals and other reactive oxygen species (ROS), which play an essential role in the pathogenesis of diseases such as asthma [4], diabetes mellitus [5], Alzheimer [6], Parkinson [7], as well as cancer [8–10]. Nanoparticles of Au, Ag, Pt or metal transition oxides such as CuO or NiO have demonstrated their antioxidant activity [11–14]. Among MNPs, gold nanoparticles (AuNPs) are one of the most studied nanoparticles due to their inert and nontoxic effects being widely used in therapeutic and diagnostic applications [15]. Several AuNPs with antioxidant capacity have been synthesized through different synthetic methodologies [3, 16, 17].

The possibility to decorate the MNPs with other antioxidant moieties opens the door to use them in biomedicine, for example in combination therapy with the aim to inhibit the ROS generated by oxidative stress in the body that are responsible of damage cells, proteins and DNA [18, 19]. Among the external sources of antioxidants (exogenous), the phenolic compounds extracted from plants are commonly used due to their capacity to induce metabolic changes and prevent several chronic diseases. Despite that these compounds possess a wide range of uses, several studies had shown that their bioavailability is limited due to poor intestinal absorption as well as low permeability in cells [20–22]. Hence, the development of new conjugated systems is necessary to overcome these problems [23–25]. One of these examples is the antioxidant activity of AuNPs functionalized with Trolox trough Au-S bound that were evaluated by 2,2-diphenyl-1-picrylhydrazyl (DPPH) radical scavenging assay showing an 8-fold activity increase in comparison with free Trolox [26]. On the other hand, the antioxidant capacity of AuNPs functionalized with natural polyphenols such as epigallocatechin-3-gallate, resveratrol and fisetin was evaluated by DPPH and 2,2′-azinobis(3-ethylbenzothiazoline-6-sulphonic acid) cation (ABTS) radical scavenging assay showing a correlation between the activity and the content of polyphenol in the conjugated AuNPs [27].

Among these antioxidants, caffeic acid (CA) and its derivatives have also shown not only antioxidant, but also antibacterial, anti-inflammatory and anticancer activities [28–32]. Also, AgNPs functionalized with CA have also been used for the treatment of osteoarthritis [33] and human hepatoma therapy [34].

Our research group has developed several dendronized carbosilane ionic AuNPs homo- and heterofunctionalized with promising biomedical applications. So, dendronized AuNPs with sulphonate peripheral groups were capable to inhibit both R5 and X4 tropism isolates of HIV-1 virus [35]. Additionally, cationic dendronized AuNPs have shown activity as antibacterial and antifungal agents [36]. Recent studies focus in heterofunctionalization of MNPs with polyethylene (glycol) (PEG) that allows the obtention of systems with higher stability, biocompatibility and permeability [37, 38]. Dendronized carbosilane AuNPs functionalized with PEG have allowed improving the biological properties of the systems reducing haemolysis as well as platelet aggregation [39].

The aim of this work is to obtain new AuNPs decorated with CA and explore their antioxidant and antibacterial properties. Herein, we report new homofunctionalized AuNPs capped with CA possessing a PEG thiol function (HS-PEG-CA) and heterofunctionalized AuNPs with HS-PEG-CA and cationic carbosilane dendrons. The presence of PEG increases the hydrophilic nature of nanoparticles improving their dispersibility in water. The antioxidant and antibacterial properties of these new derivatives have been proved through different techniques.

## 2. Materials and Methods

### 2.1. Synthesis and Characterization of Compounds

#### 2.1.1. General Conditions

All reactions were performed under an inert atmosphere, and solvents used were bought in dry conditions. NMR spectra were recorded in a Varian 500 Hz or Bruker Neo Advance 400 Hz spectrometers using CD_3_OD or CDCl_3_ as a solvent. Chemical shifts (*δ*) are given in ppm. HSQC, HMBC and COSY NMR experiments have been performed to assign the corresponding resonances signals. LECO CHNS-932 instrument was used to carry out the elemental analysis measurements. UV–visible spectra of diluted compounds samples in a quartz cell with a path length of 1 cm were recorded using a Perkin-Elmer Lambda 18 spectrophotometer. Compounds HS-PEG2K-NH_2_·HCl, 2,2-dimethoxy-2-phenylacetophenone (DMPA), HAuCl_4_·3H_2_O, 1-ethyl-3-(3-dimethylaminopropyl)carbodiimide (EDCI·HCl), hydroxybenzotriazole (HOBt), NaBH_4_ and CA were obtained from commercial sources. Compound HS-G1-NMe_2_Cl was synthesized as previously reported [40].

#### 2.1.2. Transmission Electron Microscopy (TEM)

A carbon-coated copper grid (400 mesh) covered with a drop of a diluted solution of compounds was dried. Afterwards, TEM images were recorded using a transmission electron microscope JEM 2100HT. ImageJ program was used to measure nanoparticle sizes.

#### 2.1.3. Thermogravimetric Analysis (TGA)

To perform TGA measurements, pure and dry samples of compounds (2–10 mg) in a platinum sample holder under nitrogen atmosphere were heated from 25°C to 1000°C (10°C per minute) and the spectra were recorded with a TA TGA55 (TA instrument).

#### 2.1.4. Zeta Potential (ZP)

Zetasizer Nano ZS instrument (Malvern Instruments Ltd., UK) was used to carry out the ZP experiments. Compound solution in deionized water (1 mg/mL) were filtered through a 0.22-mm syringe filter and measured in disposable Malvern plastic cuvette.

#### 2.1.5. Dynamic Light Scattering (DLS)

Malvern Zetasizer Nano ZS (Malvern Instruments) equipped with a noninvasive backscatter optics (NBS) instrument was used for the hydrodynamic diameter measurements of filtered compound solutions in deionized water (1 mg/mL). The experiments were carried out at 25°C with a previous equilibration measure (typically 5 min), and the result was expressed as a mean of at least three measurements per sample.

#### 2.1.6. Synthesis of HS-PEG2K-CA (1)

The activation of CA was the first step for synthesizing compound **1**. To accomplish this, a DMF solution of CA (41.03 mg, 0.300 mmol) was added to a mixture of EDCI·HCl (57.51 mg, 0.300 mmol) and HOBt (40.53 mg, 0.300 mmol) in DMF under an inert atmosphere. The reaction mixture remained under stirring over 1 hour. Afterwards, a DMF solution mixture of HS-PEG2K-NH_2_·HCl (300 mg, 0.150 mmol) and triethylamine (15.19 mg, 0.150 mmol) was added dropwise at 0°C under stirring and maintained for 15 min under these conditions. Finally, the reaction mixture was stirred at 60°C overnight. After purification by size exclusion chromatography in acetone, compound **1** was obtained as a yellow solid (288.5 mg, 90.2%). ^1^H-NMR (CD_3_OD): *δ* (ppm) 2.86 (m, 2 H, SC*H*_2_CH_2_O), 3.69 (m, 4 H, C*H*2C*H*_2_NH), 3.69 (m, C*H*_2_O*CH*_2_, PEG), 3.88 (m, 2 H, SCH_2_C*H*_2_O), 6.42 (d, 1 H, ^3^J_(H-H)_ = 15.7 Hz, PhCH=C*H*(CO)NH), 6.78 (d, 1 H, ^3^J_(H-H)_ = 8.2 Hz, 1 *H*_*Ar*_, meta-CH=CH), 6.92 (dd, 1 H, ^3^J_(H-H)_ = 8.2 Hz, ^5^J_(H-H)_ = 1.8 Hz, 1 *H*_*Ar*_, ortho-CH=CH), 7.02 (d, 1 H, ^5^J_(H-H)_ = 1.8 Hz, 1 *H*_*Ar*_, ortho-CH=CH, ortho-OH), 7.39 (d, 1 H, ^3^J_(H-H)_ = 15.7 Hz, PhC*H*=CH(CO)NH). ^13^C {^1^H}-NMR (CD_3_OD): *δ* (ppm) 39.6 (S*C*H_2_CH_2_O), 40.6 (*C*H_2_NH), 70.5–71.6 (*C*H_2_O*C*H_2_, PEG), 115.1 (*C*Ar, ortho-OH, ortho-CH=CH), 116.5 (*C*Ar, meta-CH=CH), 118.6 (PhCH=*C*H(CO)NH), 122.2, (*C*Ar, ortho-CH=CH), 128.3 (*C*ipso, meta-OH, para-OH), 142.2 (Ph*C*H=CH(CO)NH), 146.8 (*C*ipso, meta-CH=CH), 148.8 (*C*ipso, para-CH=CH), 169.2 (NH*C*=*O*). Elemental Analysis (%): Calc for C_97_H_185_NO_46_S (2132,19 g/mol). C, 54.61; H, 8.74; N, 0.66; S, 1.50. Exp.: C, 54.54; H, 8.63; N, 0.91; S, 1.40.

#### 2.1.7. Synthesis of AuNPs(S-PEG2K-CA) (2)

To a deoxygenated aqueous solution of HAuCl_4_ (1.56 mL, 0.047 mmol, 30 mM, 15.93 mg) was added dropwise an aqueous solution of compound **1** (3.75 mL, 0.047 mmol, 12.5 mM, 100 mg). Afterwards, NaBH_4_ in deoxygenated water (1.17 mL, 0.234 mmol, 200 mM, 8.87 mg) was added dropwise, and the mixture remained under stirring over 4 h. AuNPs were purified by dialysis (MWCO: 20,000 Da) yielding **2** as a grey solid dispersed in water. The synthesis was carried out in triplicate. Mean diameter of gold core (TEM) was 1.0 nm. The results are expressed as mean ± SEM: ZP (mV): +1.10 ± 1.34. DLS (Z-average diameter, nm): 75.91 ± 3.09.

#### 2.1.8. Synthesis of AuNPs(S-PEG2K-CA) (S-G1-NMe3Cl)

Deoxygenated aqueous solutions (2.2 mL, 12.5 mM) of a mixture of compound **1** (50 mg, 0.023 mmol) and HS-G1-NMe_3_Cl (10.01 mg, 0.023 mmol) were added dropwise to a deoxygenated water solution of HAuCl_4_ (0.78 mL, 0.027 mmol, 30 mM, 10.71 mg). Then, a deoxygenated water solution of NaBH_4_ (0.59 mL, 0.117 mmol, 200 mM, 4.44 mg) was added dropwise, and the reaction mixture was stirred for 4 h. Purification by dialysis (MWCO: 20,000 Da) was allowed to obtain compound **3** as a black solid highly soluble in water. The synthesis was carried out in triplicate. The results are expressed as mean ± SEM: ZP (mV): +14.4 ± 1.36 DLS (Z-average diameter, nm): 53.61 ± 3.62.

### 2.2. Antioxidant Activity

#### 2.2.1. DPPH Radical Scavenging Activity

Antioxidant capacity was evaluated by the ability of compounds to scavenge DPPH radical. Aliquots of 180 μL of DPPH methanolic solution (111.11 μM) were placed in 96-well plates. Afterwards, 20 μL of the compounds in water at concentrations ranging from 0.8 to 60 μg/mL (homofunctionalized) and 10 to 120 μg/mL (heterofuncionalized) was added and the well plate was kept in the dark at room temperature for 30 min. After that period, the absorbance was recorded at 530 nm using a microplate reader (Epoch™, BioTek Instruments, Winooski, VT, USA). All tests were performed in triplicate, and methanol was used as control. The scavenging activity was determined based on the reduction of DPPH absorbance. IC_50_ (concentration that produces 50% of antioxidant activity) was calculated. For this purpose, it was necessary to calculate the DPPH remnant.

#### 2.2.2. FRAP

Antioxidant activity was also evaluated by the ability of compounds to reduce Fe^+3^ to Fe^+2^ in the presence of 2, 4, 6-tripyridyl-striazine (TPTZ), forming an intense blue Fe^+2^–TPTZ complex with maximum absorption at 593 nm. Aliquots of 180 μL of FRAP solution (mixture of solutions TPTZ (10 mM solution in 40 mM of hydrochloric acid), FeCl_3_ (20 mM solution in ACS water) and acetate buffer (20 mM in ACS water, pH 3.6) in a 1:1:10 ratio) were placed in 96-well plates. Afterwards, 20 μL of the compounds in water at concentrations ranging from 0.01 to 0.6 μg/mL was added, and the well plate was kept in the dark at room temperature for 30 min. After that period, the absorbance was recorded at 593 nm microplate reader (Epoch™, BioTek Instruments, Winooski, VT, USA). All tests were performed in triplicate, and water was used as control. Finally, EC_50_ (concentration of antioxidant compound that increases 50% of FRAP capacity) was calculated.

#### 2.2.3. Electrochemical Measurements

Electrochemical measurements were carried out on an Autolab PGSTAT 204 potentiostat from Metrohm (Utrecht, The Netherlands), employing screen-printed carbon electrodes (SPCEs, DRP-110, *ϕ* 4 mm, real area 0.05 cm^2^). Both working (WE) and counter (CE) electrodes are made of carbon, and Ag/AgCl ink as the reference electrode. Depending on the measurement conditions, different concentrations of each compound (HS-PEG2K-CA (**1**), AuNPs(S-PEG2K-CA) (**2**), and AuNPs(S-PEG2K-CA) (S-G1-NMe_3_Cl) (**3**)) were studied in phosphate buffer (PBS, 0.1 M, pH = 7.4).

The reductive desorption (RD) experiment was carried out as follows. A self-assembled monolayer of the L molecule was formed by contacting a homemade polyoriented gold substrate (po-Au) with 1.0 mM of ligand in PBS 0.1 M solution. To ensure the formation of a uniform, compact SAM, 16 h was selected as modification time. After such time, the modified electrode was thoroughly washed with PBS 0.1 M and water and then dried under a nitrogen stream. The RD process of the L could be hindered by the presence of oxygen in the solution. Thus, before the experiment, we eliminated the dissolved oxygen by purging the solution of the electrochemical cell (KOH 0.1 M) with an inert gas such as nitrogen.

Calculation of the equivalent concentration of CA moieties on homo- (2), and heterofunctionalized (3) AuNPs: Based on TGA results, each homofunctionalized AuNP contains 15 ligand molecules. A colloidal suspension of 1 g/L (total amount of mass) is analysed and, from that, samples that contain a total of 3.8·10-4 M CA are prepared to be evaluated by using SWV.

### 2.3. Antibacterial Activity

The experiments were carried out in Chemistry Research Support Centre of the University of Alcala. In vitro antibacterial analysis of dendritic polyphenols was performed following the international standard method ISO 20776-1:2006. The antibacterial activity was assayed in two different bacteria, *Escherichia coli* (CECT 515) and *Staphylococcus aureus* (CECT 240). For this purpose, stock solutions in water of each compound were made and then, subsequent dilutions in Mueller–Hinton agar (Scharlau, ref. 02–136) were developed to obtain the testing concentrations (from 0.25 to 1024 ppm). The assay was carried out in 96-well plates, with two different wells for each concentration and with different controls (biocide, inoculum and culture medium). The bacteria were inoculated at a concentration of 10^7^ CFU/mL in wells. After 24 h of incubation with biocides at 37°C, the increase of turbidity at 630 nm was determined using an Ultra Microplate reader (BIO-TECK Instruments, model epoch 2). MIC and MBC values were obtained.

## 3. Results and Discussion

### 3.1. Synthesis and Characterization of Gold Nanoparticles

**Scheme 1:**
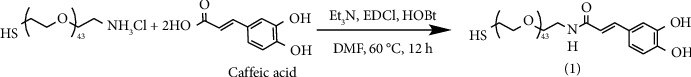
Synthesis of HS-PEG2K-CA derivate **1**.

Since the presence of PEG units in the nanoparticles enhances the dispersibility of them in aqueous solutions, the first step in this study was the derivatization of the commercial thiol HS-PEG2K-NH_2_·HCl. This compound allows the introduction of CA through an amidation coupling reaction with EDCI·HCl and HOBt in basic condition (Scheme 1). The thiol group would make possible later to obtain functionalized gold nanoparticles via direct thiol-capping reaction.

To achieve the synthesis of HS-PEG2K-CA (**1**) derivate, a protocol previously developed in our research group, with some modifications, was followed [41]. Afterwards, the purification by size exclusion chromatography leads compound **1** as a yellow solid in high yields.

**Figure 1:**
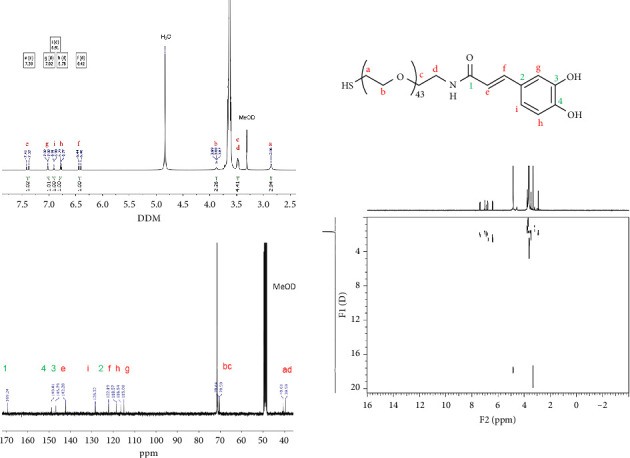
NMR spectra in CD_3_OD of HS-PEG2K-CA derivate (**1**): (a) ^1^H-NMR, (b) ^13^C-NMR and (c) ^1^H-DOSY-2D-NMR. (d, doublet; dd, doublet of doublets).

A ^1^H-NMR, ^13^C-NMR and ^1^H-DOSY-2D-NMR experiments were used to confirm the formation of the desired HS-PEG2K-CA derivate (Figure 1). The NMR data of compound **1** corroborated the formation of an amide bond by the appearance of the signals corresponding to the methylene group bonded to the amide nitrogen at 3.69 ppm in the ^1^H-NMR and 40.6 ppm in ^13^C-NMR, as well as the signal of the carbonyl group at 169.2 ppm in ^13^C-NMR. Furthermore, signals assigned to the alkene fragment at 6.42 and 7.39 ppm in ^1^H-NMR and 118.6 and 142.2 ppm in ^13^C-NMR along with a set of aromatic signals at 6.78, 6.92 and 7.02 ppm in ^1^H-NMR and between 115.0 and 148.9 ppm in ^13^C-NMR were observed. In addition, Elman's test that allows determining the presence of free thiol groups was performed confirming the presence of this group in the compound [42]. ^1^H-DOSY-2D-NMR experiments were performed, allowing to corroborate the presence and purity of the corresponding compound.

**Scheme 2:**
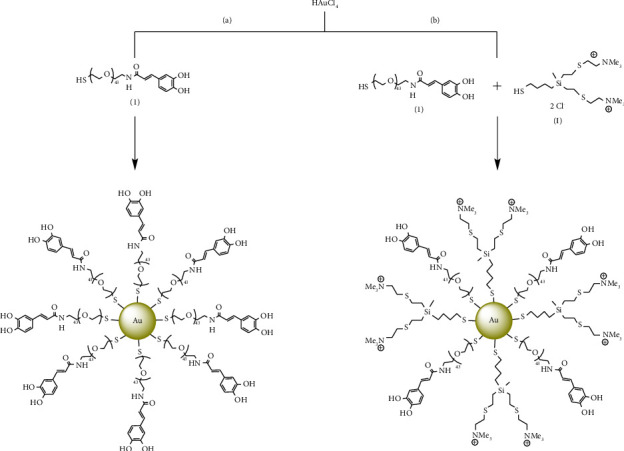
Synthesis of (a) AuNPs(S-PEG2K-CA) (**2**) and (b) AuNPs(S-PEG2K-CA) (S-G_1_NMe_3_Cl) (**3**).

After obtaining the thiol-caffeic derivative, gold nanoparticles were synthesized. In this work, our focus has been on the preparation of two types of nanoparticles (a) AuNP homofunctionalized with CA in their surface and (b) AuNP heterofunctionalized in which the NP surface, besides containing some units of caffeic derivates, presents carbosilane dendrons with ammonium groups at the periphery. In both cases, the synthesis was carried out according to a method previously developed by our research group [36, 43]. For homofunctionalized NP, the reaction was carried in an aqueous medium where the precursor of gold ions HAuCl_4_ was reduced by NaBH_4_ in the presence of the stabilizing ligand **1** (Scheme 2(a)). The purification by dialysis (molecular weight cut-off (MWCO): 10 kDa) allowed the obtention of the compound **2** (AuNPs(S-PEG2K-CA)) as a grey solid dispersed in water. Despite the presence of PEG ligand, these new systems did not form stable dispersions in water and precipitated over time.

The new heterofunctionalized AuNPs were obtained through a previously described protocol where the main difference was the presence of two capping ligands in the reaction (Scheme 2(b)) [37]. In this case, cationic carbosilane dendron of first-generation [40] (HS-G_1_NMe_3_Cl) with a thiol group at the focal point was used as a second capping ligand. Heterofunctionalized AuNPs(S-PEG2K-CA) (S-G_1_NMe_3_Cl) (**3**) were obtained after dialysis purification (MWCO: 10 kDa) as a black solid forming highly stable dispersions in water. The presence of cationic dendron improves the solubility of the NPs, overcoming the problem of dispersion stability in aqueous media found homo AuNP (**2**). In addition, the introduction of the cationic carbosilane dendrons could expand its possible biomedical application by a cooperative effect with the phenolic ligand, since the presence of these two functional groups can provide antioxidant and antibacterial activity simultaneously.

^1^H-NMR spectra of homofunctionalized AuNPs(S-PEG2K-CA) (**2**) show the signals corresponding to S-PEG2K-CA ligands even though those corresponding to the CA fragment do not appear; this can be attributed to a change in the structure of the ligands as well as their proximity to the metal core due to a possible backfolding of the PEG ligands [44]. In respect of AuNPs(S-PEG2K-CA) (S-G_1_NMe_3_Cl) (**3**), a signal corresponding to PEG was observed but, unfortunately, those corresponding to the carbosilane dendron were broad and again could be attributed to the proximity of dendrons to the AuNP surface.

The dimensions, charge and stability over time of synthesized NP were studied through diverse techniques such as TEM, DLS, TGA, ^1^H-NMR spectroscopy and ZP were employed.

**Figure 2:**
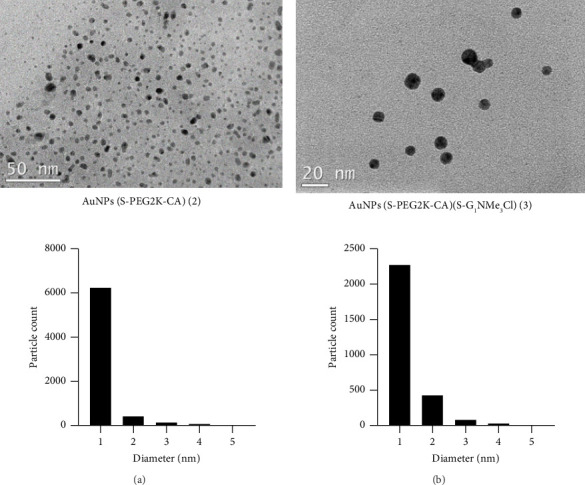
TEM image and size distribution histogram of (a) AuNPs(S-PEG2K-CA) (**2**) and (b) AuNPs(S-PEG2K-CA) (S-G_1_NMe_3_Cl) (**3**).

For determining the morphology and sizes of both AuNPs, DLS measurements were compared with TEM studies. TEM images of AuNPs synthesized in this study confirm the formation of the desired compounds observing the same mean diameter for both homo- (**2**) and heterofunctionalized (**3**) AuNPs. The results obtained demonstrated that AuNPs' sizes are not affected using one or two capping ligands where most nanoparticles were about 1 nm in diameter (Figure 2). To evaluate the colloidal stability of the nanoparticles, complementary experiments of DLS were performed in solutions. DLS measurements based on the analyses of the scattered light intensity fluctuations from the Brownian motion of the particles dispersed in a liquid allow the calculation of diffusion coefficients and the hydrodynamic size of the particles by Stokes–Einstein equation [45]. In this study, the hydrodynamic size was first evaluated at short term using volume-weighted distributions to characterize the primary dispersion state. Long-term aggregation behaviour was subsequently studied using intensity-based distributions, which are particularly sensitive to the presence of larger aggregates. The obtained result at short term presented in terms of volume confirms that the main population is centred around the size 8.25 ± 1.91 nm of heterofunctionalized AuNPs (**3**) and the size 7.71 ± 1.35 nm homofunctionalized AuNPs (**2**). The obtained long-term results using the intensity distribution display the presence of agglomerates and aggregates with slight decrease in size in the case of AuNPs (**3**) observing nanoparticles with a mean diameter of 53.61 ± 3.62 (Z-average diameter, nm), while AuNPs (**2**) display a mean diameter of 75.91 ± 3.09 (Z-average diameter, nm). This may be because of a lower number of CA molecules on the surface of NP-**3**, which reduces the possibilities of formation of aggregates by intermolecular hydrogen bonding interaction. However, the differences observed between the TEM and DLS measurements, higher diameter obtained by DLS in comparison with TEM, can be explained by considering that in TEM the size corresponds to the measurement of the ‘shadow' or image projection defined by the metal core, while DLS determines the hydrodynamic diameter of nanoparticles in suspension, encompassing not only the core particle size but also contributions arising from particle aggregation or agglomeration within the colloidal system, as well as the presence of an electrical double layer at the interference between the nanoparticle surface and the surrounding medium. The size of the electrical double layer and thus the hydrodynamic radius are modified by changes in the surface structure of the nanoparticle and/or the electrolyte concentration of the medium. Also, as previously mentioned above DLS determines not only the particle size but also the presence of agglomerates and aggregates and thus assesses their stability and polydispersity (PDI). In this study, gold nanoparticles were modified with PEG containing a single-terminal thiol (SH) group, as illustrated in Scheme 2. The PEG used had a molecular weight of 2000 (PEG2000), which, due to its relatively short chain length, would be expected to contribute only modestly to the overall particle diameter. However, when grafted onto the nanoparticle surface, PEG chains can increase the hydrodynamic size by more than a few nanometres, depending on their conformation in aqueous solution and the surface grafting density. The DLS results reflect not only the PEG-induced surface modification but also the limited dispersion state of the nanoparticles in solution, which likely promotes partial aggregation—an effect readily detected by DLS due to its sensitivity to larger scattering species. This type of size discrepancy between DLS and TEM measurements has also been observed previously by our research group in systems using PEG-coated gold nanoparticles with varying dendron/PEG ratios, where PEG of molecular weight 800 was employed [37, 39]. An estimation of the theoretical core diameter of the AuNPs was performed using the PDI of DLS (*D*_*x*_/(1 + PDI)^5^) [40, 46]. The results of this analysis are reported in the Table S1 of the supporting information.

Regarding ZP, measurements of aqueous solutions of AuNPs at neutral pH show a +1.10 ± 1.34 mV for homofunctionalized AuNPs (**2**) to + 14.4 ± 1.36 mV for heterofunctionalized AuNPs (**3**). This behaviour can be explained by the presence of a positive charge in cationic carbosilane dendrons of heterofunctionalized AuNPs. Nevertheless, this value of ZP is significantly lower than those observed for AuNPs homofunctionalized with cationic carbosilane dendrons as a capping ligand [40]. This dramatic decrease can be explained by a probable charge cover by the S-PEG2K-CA ligands.

**Figure 3:**
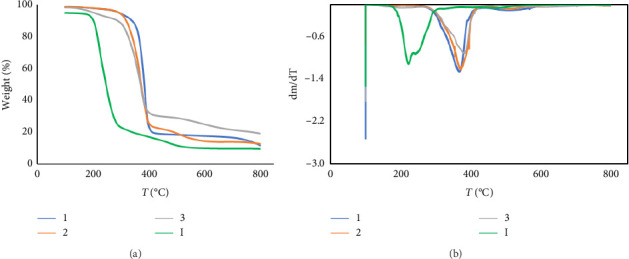
Graphics of (a) weight loss in function of temperature and (b) first derivative of the weight loss for capping ligands (HS-G_1_NMe_3_Cl (**I**) and HS-PEG2K-CA (**1**)) and AuNPs (AuNPs-SG_1_NMe_3_Cl (**II**), AuNPs(S-PEG2K-CA) (**2**) and AuNPs((S-PEG2K-CA) (S-G_1_-NMe_3_Cl) (**3**)).

Finally, TGA was carried out to determine the percentage of organic matter coating the metallic core. For this purpose, completely dry AuNPs **2** and **3**, as well as free capping ligands HS-PEG2K-CA (**1**) and HS-G_1_NMe_3_Cl (**I**), were heated from 25°Cto 1000°C under N_2_. The weight loss observed during the heating process corresponds to the degradation of organic matter in the compound. In the case of free thiols, the degradation temperature for cationic carbosilane dendron HS-NMe_3_Cl (**I**) was between 200°C and 300°C; meanwhile, HS-PEG2K-CA (**1**) degrades at higher temperatures (300°C–600°C) (Figure 3(a)).

A comparison of the data obtained from the first derivative of weight loss as a function of temperature confirms the presence of the capping ligands on AuNPs (Figure 3(b)). The weight loss between 300°C and 600°C corresponding to thermal decomposition of S-PEG2K-CA capping ligands was observed for both homo- (**2**) and heterofunctionalized (**3**) AuNPs, while weight loss between 100°C and 300°C due to carbosilane dendron S-G_1_NMe_3_Cl capping ligand thermal decomposition was only observed in the case of heterofunctionalized (**3**) AuNPs.

The number of ligands anchored to each AuNP was estimated based on the results achieved (see Figure S1).  Table 1 shows the results obtained considering the nanoparticle average diameter core obtained by TEM (1.0 nm diameter), which allows to determine the gold atoms (N_Au_) in AuNPs **2** and **3** core, assuming spherical nanoparticle shape and considering the weight loss contained by TGA. Finally, taking into account the core area of the AuNP, it was possible to estimate the average surface coverage (Γ). (See supporting info).

### 3.2. Antioxidant Capacity

It is well known that antioxidant capacity could be different depending on the reaction mechanism of the assay used [47]. In this study, to establish the antioxidant capacity of the compounds, several assays with different antioxidant mechanisms were used. On the one hand, two spectrophotometric methods were used, DPPH radical scavenging assay which involves a single-electron transference (SET) and hydrogen atom transference (HAT) mechanisms and FRAP assays that only involve a SET mechanism. On the other hand, electrochemical assays of cyclic voltammetry (CV) were also used to test the antioxidant activity of the new synthesized compounds.

#### 3.2.1. Spectrophotometric Assays

Both assays, DPPH and FRAP, are valuable tools for assessing the antioxidant potential of natural compounds, food products and pharmaceuticals to determine the free radical scavenging abilities and reducing power of them, helping in understanding their potential health benefits and applications.

**Figure 4:**
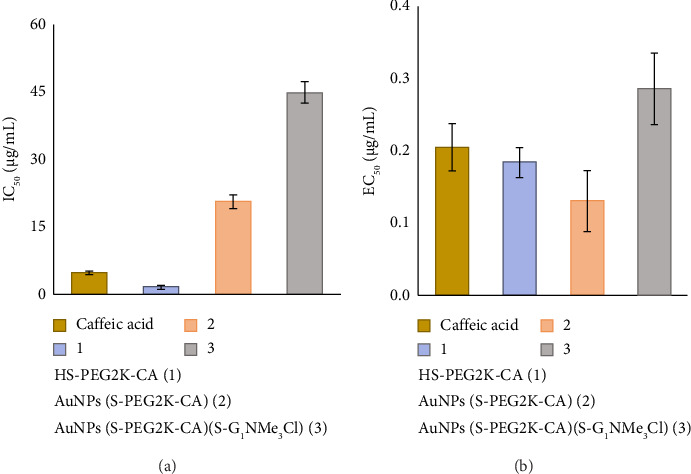
Antioxidant activity of compounds: (a) IC_50_ in DPPH assay, (b) EC_50_ in FRAP assay.

Interpretation of results, showed in Figure 4, was performed from two different perspectives, evaluating IC50 (concentration of antioxidant dendrimer that can remove 50% of DPPH free radical) and EC50 (concentration of antioxidant dendrimer that can increase 50% of FRAP capacity).

Analysis of the data obtained by DPPH (see Figure S1) showed that compound **1** exhibited an IC_50_ value of 1.607 μg/mL and excellent response as a DPPH radical scavenger, even showing a higher activity than free CA. As expected, nanoparticle **2**, which contains only derivative **1** in its structure, was more active (IC_50_ value of 20.655 μg/mL) than nanoparticle **3** (IC_50_ value of 45.025 μg/mL), which contains, in addition to ligand **1**, part of the structure functionalized with the cationic carbosilane dendritic wedge.

In the analysis of the antioxidative capacity of the systems by FRAP (ability to reduce iron(III) to iron(II) by a one-electron transfer reaction, see Figure S2), again the heterofunctionalized nanoparticle was the least active (EC_50_ value of 0.285 μg/mL). However, nanoparticle **2** (EC_50_ value of 0.1311 μg/mL) was found to be slightly more active than derivative **1** (EC_50_ value of 0.183 μg/mL) and free CA. The combination of the data obtained by both methods indicates that the nanoparticles obtained maintain the antioxidant activity of CA when it is supported on the surface of the nanoparticle.

#### 3.2.2. Electrochemical Characterization

**Figure 5:**
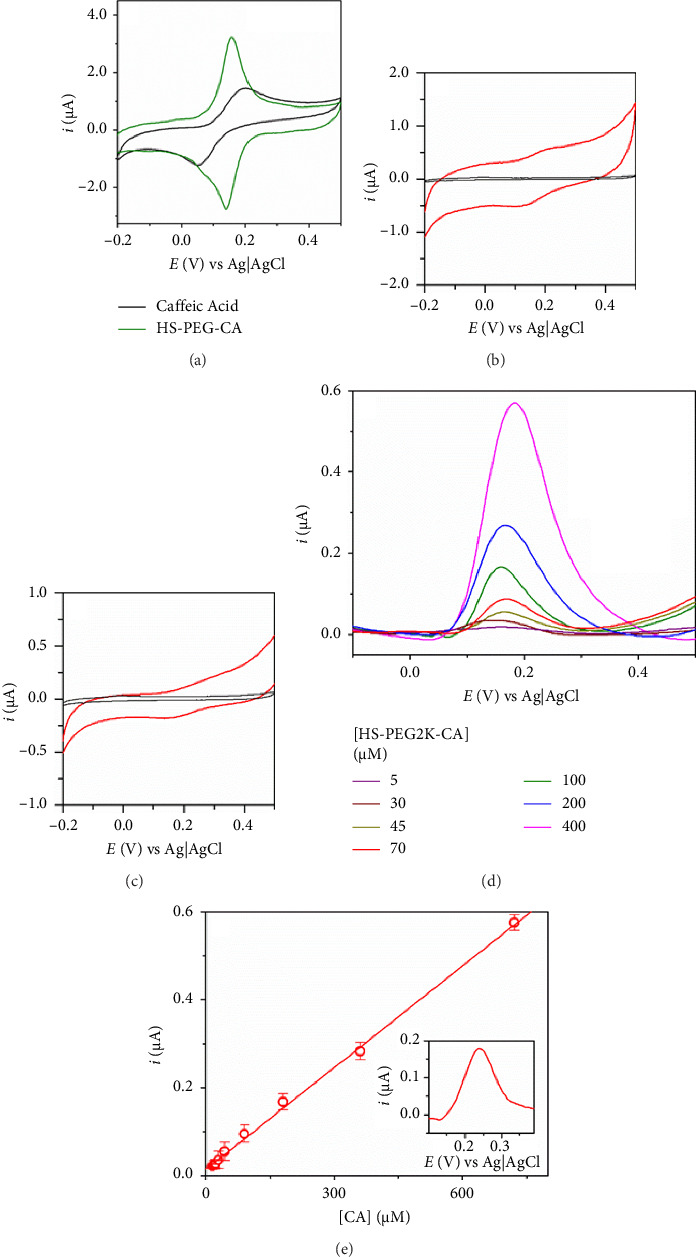
Cyclic voltammograms of free HS-PEG2K-CA **(1)** (a), and the corresponding homo- **(2)** (b) and heterofunctionalized **(3)** (c) AuNPs. CV experiments were carried out at 50 mV s^−1^ in phosphate buffer (pH = 7.4, 0.1 M). All voltammograms were recorded in the presence of an equivalent concentration of 10 μM CA in each molecule (red signal). The black line corresponds to the background scan. (d) SWV signals obtained from different concentrations of the free ligand, **1**. (e) Calibration using the peak intensities obtained from D. Inset: Concentration determination of CA equivalents in a sample containing 40 μM of homofunctionalized AuNPs.

*CV and SWV behaviour of CA moieties:* The electrochemical performance of the thiolate ligand (**1**), the synthesized homo- (**2**) and hetero- (**3**) functionalized AuNPs, as well as the free CA, was investigated using CV and square wave voltammetry (SWV). It is well known that the redox couple implicated in the electro-oxidation process of CA at neutral and acid pH involves the electron transfer of two electrons (Scheme S1). CV characteristic of **1**, and AuNP **2** and **3** are presented in Figure 5. CV is usually employed to study the ability of a molecule to perform electron transfer and, thus, its antioxidant activity can be estimated by the analysis of the anodic peak potential (*E*_pa_) position, intensity (*i*_pa_) and peak-to-peak separation with respect to the cathodic peak (*E*_pc_) position (Δ*E*_*p*_, reversibility) [48, 49]. More specifically, higher antioxidant activity is directly related to an enhancement of the heterogeneous electron transfer rate constant (HET) (*k*^0^). These parameters have been determined for the species under investigation and are summarized in Table 2.

They have been extracted from the analysis of the electrochemical response of free CA, the thiolate ligand (**1**), homo-(**2**), and hetero-(**3**) functionalized AuNPs. Since the redox behaviour is supported by CA moiety of the ligands only, we have used equivalent concentrations of CA (10 μM) to record the CVs presented in Figure 4. To determine this equivalent concentration, we considered the results obtained from the TGA analysis (Table 1). The number of ligands present on the nanoparticle is calculated using the Au/ligand ratio, obtained from the above-mentioned analysis: The weight loss in the different range of temperatures is related to the nature of the different capping ligands that degrade and their corresponding molecular weight allowed calculating the quantity of each capping ligand grafted to AuNPs. Details of the calculations are presented in the Supporting Information. All the systems show quasi-reversible behaviour. However, the differences in peak-to-peak potential separation are remarkable. Cyclic voltammograms obtained for free CA present narrow, well-defined peaks, with the oxidation one positioned at ca. 0.194 V, and a Δ*E* of 240 mV. Interestingly, based on the CVs present in Figures 5(a), 5(b), 5(c), systems **1**, **2** and **3** prepared exhibit an improvement in the reversibility of the redox pair, which is evidenced by a decrease in Δ*E*. The free ligand **1** presents an excellent response, and in fact, the decrease in Δ*E* is accompanied by an increase in the peak intensity. Based on the CVs present in Figures 5(b), 5(c), we can ensure that CA peaks are present in **2**- and **3**-AuNPs. However, the recorded signal is quite poor, especially in the heterofunctionalized AuNPs. The observed results suggest that the functionalization of the AuNPs hinders the electrochemical transfer to the electrode. In the case of the hetero-AuNP **3**, such hindrance is accentuated as there are other sorts of molecular chains on the surface (S-G1-NMe_3_Cl) that do not present an electrochemical response. Despite that, we can conclude that anchoring the CA molecule to the thiolate PEG chain facilitates the electron transfer of the first one.

To facilitate the discussion, CVs carried out at the same concentration of CA present in different systems (free CA molecule and CA moieties in **1**, **2** and **3**) have been plotted together in Figure S3. If the intensity of the signals recorded for **2**- and **3**-AuNPs is compared, it could be thought that only part of the CA moieties present takes an active role in the electrochemical transfer. This can be due to the interfacial nature of the electrochemical transfer over the electrode, and the size of the homofunctionalized AuNPs **2** can cause a steric hindrance to the further moieties from the electrode [50]. On the other hand, it is surprising to find that the signal of ligand, **1**, is even better than that of the free CA. The observed response suggests that the PEG contributes in some way to the enhancement of the ET of the electroactive part of the molecule. Such behaviour might be explained by the interaction of the oxygen atoms in the backbone, which results in high-lying, delocalized molecular orbitals that appear from superexchange coupling between adjacent lone-pair orbitals on oxygen [51].

To deeply investigate the above-mentioned phenomena, known concentration of hetero- and homofunctionalized AuNPs was measured using SWV, and the obtained current was interpolated in a standard calibration using the free ligand **1**. By using this strategy, we can evaluate the amount of CA moieties in **2** and **3** that are able to show electrochemical responses once anchored to the AuNPs. Figure 5 shows SWV signals obtained from different concentrations of CA moieties of HS-PEG2K-CA (D), and the corresponding calibration curve (E). We used samples of **1**, **2** and **3**, which contain an equivalent concentration of CA of ca. 400 μM. As expected, the interpolation of sample **1** in the calibration plot gave us an equivalent concentration of ca. 400 μM. However, the interpolation of **2** and **3** SWV responses results in ca. 200 and 50 μM, respectively (Table 3). The results showed that not all the CA units present in the nanoparticle are capable of producing an electrochemical response, perhaps due to a backfolding phenomenon on the surface of the nanoparticle, which hinders exposure to the electrode. This effect is more pronounced when the nanoparticle is also heterofunctionalized with the dendritic wedge of carbosilane nature.

##### 3.2.2.1. Calculus of Diffusion Coefficient (*D*_0_) and HET (*k*^0^)

The intramolecular electrochemical enhancement of CA performance inside the SH-PEG2K-CA molecular structure, and anchored to AuNPs, is evidenced by the determination of the diffusion coefficient (*D*_0_) and the HET (*k*^0^). Both parameters can be obtained from the study of electroactive species in solution as a function of the scan rate in CV (Figure S4).

We have successfully assessed the CA, **1**, and homofunctionalized AuNPs (Table 3). In the case of hetero-AuNPs **3**, the signal recorded as a function of the scan rate is very weak and quickly becomes characteristic of an irreversible process, even at intermediate/low scan rates, making it impossible to accurately determine *D*_0_ nor *k*^0^. The first is obtained from the Randles–Sevcik equation, and the second, by using the Nicholson method [52, 53]. Details about these methods are described in the *Supporting Information*.

In general terms, higher diffusion coefficients are related to an easier movement of the reagents through the solution. The derivate **1** has the highest value of *D*_0_ (2.2 ± 0.2)·10^−4^ cm^2^ s^−1^. This could indicate that the presence of the pegylated chain somehow catalyses the electronic transfer of the CA moiety of the molecular chain. The improved electron transfer in the free ligand and the homofunctionalized nanoparticles, as compared to CA, was also confirmed using the Nicholson method. Like what was shown for the D_o_ value, the unbound pegylated molecule has the fastest electron transport, with a high value of *k*^0^ (2.8 ± 0.3)·10^−1^ cm s^−1^. The anchoring of the molecular chain to the NP surface (5.8 ± 0.6)·10^−3^ cm·s^−1^ restricts the freedom of movement of the electroactive part. However, compared to the free CA molecule (1.6 ± 0.2)·10^−3^ cm·s^−1^, an improvement in the rate of the electron transfer process is discernible.

##### 3.2.2.2. CA-PEG-S-Au SAM Formed on Gold Electrodes

*3.2.2.2.1. Electron Transfer Rate Constant (k*_*s*_ *) of Molecules Anchored to the Electrode Surface.* The excellent antioxidant capacity exhibited by the synthesized HS-PEG2K-CA ligand **(1)** invites us to delve into their potential as molecular coatings on flat metal substrates.

By anchoring the ligand molecule **1** to the electrode surface, we confine the electronic transfer process of the redox couple from the CA moiety to the surface. The process that occurs is known as mass transport and is diffusive in nature. In surface-confined organized systems, it is possible to determine the electron transfer constant based on the equations given by Laviron's model (described in the *Supporting Information*) [54]. From the analysis of the plot of the peak potential versus logarithmic scan rate (experimental data from CV, scan rate study) (Figure S5), it is observed that at high scan rates the values of *E*_*p*_ vary linearly with log *v* and, thus, it is possible to calculate *α* and *k*_*s*_ by applying equations S2-S4. In our system, 0.5 and 18 ± 1 s^−1^ are the values obtained for *α* and *k*_*s*_, respectively. These data show that the electroactive CA group presents a fast electron transfer process, so the ligand reported in the present work could be considered in the design of molecular polymeric films for the development of electrochemical (bio)sensors.

*3.2.2.2.2. RD Process.* It is known that the RD process, monitored by CV, may provide information regarding factors such as the stability and compactness of the film as well as the surface coverage of the prepared layer. Such surface coverage from the analysis of the RD process (Figure S6) is based on the widely accepted reaction [55].

(1) $$\begin{array}{c} R-S-\text{Au}+\text{solvent}+1e^{-}⟶\;{\text{Au}}_{\text{solvent}}+R-{S_{\text{solvent}}}^{-}. \end{array}$$

Thus, the charge involved in the RD process can be employed to estimate the surface coverage, Γ, from equation Γ = *Q*/*nF*, where *Q* is the charge density (C·cm^−2^), *n* is the number of electrons involved, and *F* is the Faraday's constant (C·mol^−1^). An approximate calculation of Γ gives us a value of (5.0 ± 0.2)·10^−10^ mol cm^−2^ and an area per molecule of 34 ± 2 Å^2^. This value agrees with that reported for SAMs prepared on Au electrodes from methylene-terminal molecules containing the same number of EG units (HS-PEG2KD-CH_3_) in their structure [56].

It is interesting to compare these results with those obtained by TEM. From the analysis of TEM, the AuNP average diameter core was estimated to be 1.0 nm and, from the TGA measurements, the loss of mass corresponding to organic matter, that is, thiolate H-S-PEG-CA ligands, allows us to determine the number of chains anchored to each metallic core: 15 units. As the AuNPs employed in this work are spherical, the available surface area *per* AuNP is 314 Å^2^. Thus, an area per molecule of ca. 21 Å^2^ is calculated. This molecular coating is much higher than the determined based on RD measurements for the S-PEG2K-CA grafted onto an Au electrode. This discrepancy is related to the large curvature that the nanoparticle surface exhibits, which likely minimizes steric hindrance between the surface-anchored chains and thus allows an increase in the number of molecules that can be efficiently grafted.

### 3.3. Antibacterial Activity

Several studies have highlighted the interest in the antibacterial properties of some polyphenolic compounds, because they appear to have the ability to combat drug-resistant bacteria that are not sensitive to conventional antibiotics in three possible ways: through direct bacterial death, through synergistic activation of antibiotics and through attenuation of bacterial pathogenicity [57, 58].

In the present study, the antibacterial activity of both the synthesized gold nanoparticles **2**, **3** and compound **1** against Gram-positive (*Staphylococcus aureus)* and Gram-negative (*Escherichia coli)* bacteria was investigated. Cell wall structure, staining characteristics, antibiotic susceptibility and outer membrane composition are the major differences between Gram-positive and Gram-negative bacteria. These differences have important implications for treating bacterial infections and for developing antibiotics.

Considering that Gram-negative bacteria have a thinner layer of peptidoglycan in their cell walls, surrounded by an outer membrane composed of lipopolysaccharides (LPS), and Gram-positive bacteria have only one membrane, it is not surprising the results shown in Table 4, which shows the activity of the AuNP **2**, **3** and compound **1** for both types of bacteria. The results showed that compound **1** and homofunctionalized nanoparticle AuNP-**2,** with only S-PEG2K-CA moieties on the surface, were inactive against both strains tested. As expected, the dendritic wedge (**I**) showed high antibacterial activity (*E. coli*: MIC = 16 mg/L; MBC = 16 mg/L and *S. aureus*: MIC = 4 mg/L; MBC = 8 mg/L), more pronounced for *S. aureus* due to the different structural composition of membranes in both strains. Heterofuncionalized AuNP-**3**, as a consequence of the presence of the dendritic wedge (**I**) on its surface, showed activity against *S. aureus* (MIC = 256 mg/L; MBC = 512 mg/L), being less active than the wedge alone (MIC = 4 mg/L; MBC = 8 mg/L). This lower activity can be attributed to the fact that the analysis of the composition of the AuNP-**3**, discussed previously, indicated that the NPs contained only less than 7% of the dendron **I**.

## 4. Conclusions

In this work, gold MNPs with CA on their surface have been obtained. The synthesis of these systems was carried out after synthesizing the HS-PEG-CA ligand, which exhibits remarkable and very promising electrochemical behaviour. The evaluation of the diffusion coefficient (D0) and the HET (k0) suggests that there is an intramolecular electrochemical enhancement of CA performance within the molecular structure of the SH-PEG2K-CA ligand due to the presence of the ethylene glycol unit.

Additionally, the introduction of cationic carbosilane wedges on the surface of the polyphenolic nanoparticles increases the dispersion stability in aqueous media. It has been demonstrated that the antioxidant capacity of CA is preserved in both homo- and heterofunctionalized nanoparticles and increases when it is part of the ligand SH-PEG2K-CA. As a proof of concept, we demonstrate that the ammonium groups from the dendron confer antibacterial activity to the heterofunctionalized nanoparticles while preserving their antioxidant properties, highlighting their potential as dual-action innovative antioxidant agents with antimicrobial capacity for pharmaceutical and cosmetic applications.

## Figures and Tables

**Table 1:** Estimation of^a^ the ligands anchored to each AuNP and^b^ total surface coverage based on the results obtained from TGA analysis (% of organic matter) and TEM (1.0 nm AuNP size).

AuNP	Molecule	% TGA	L/AuNP^a^	Γ (Å^2^/molecule)^b^
2	-S-PEG2K-CA	85	15	21
3	-S-PEG2K-CA	66	7	31
	-S-G_1_-NMe_3_Cl	7	3	

**Table 2:** Electrochemical parameters obtained from the analysis of the compounds.

Compound	*E*_*a*_ (V)^a^	*i*_*a*_ (μA)^b^	Δ*E* (mV)^c^	*D*_0_ (cm^2^·s^−1^)^d^	*k* ^0^(cm·s^−1^)^e^
CA	0.19 ± 0.01	1.5 ± 0.2	240 ± 8	(3.6 ± 0.1)·10^−5^	(1.6 ± 0.1)·10^−3^
**1**	0.16 ± 0.01	3.3 ± 0.2	30 ± 1	(2.2 ± 0.1)·10^−4^	(2.8 ± 0.2)·10^−1^
**2**	0.22 ± 0.01	0.60 ± 0.08	80 ± 3	(3.5 ± 0.2)·10^−5^	(5.8 ± 0.3)·10^−3^
**3**	0.30 ± 0.02	0.24 ± 0.03	160 ± 4	—	—

^a^Anodic peak potential.

^b^Anodic intensity.

^c^Peak-to-peak separation.

^d^Diffusion coefficient.

^e^Electron transfer rate constant.

**Table 3:** Analysis of the electroactive response of caffeic acid moieties present in **1**, **2** and **3** obtained by SWV measurements.

Compound	Sample (CA) (μM)	*i*_*a*_ (μA)	Equivalent (CA) (μM)	% Electroactive CA molecules
HS-PEG_2KD_-CA (1)	405 ± 1	0.32 ± 0.01	399 ± 1	100
AuNPs(S-PEG_2KD_-CA) (2)	412 ± 2	0.16 ± 0.04	195 ± 3	50
AuNPs(S-PEG_2KD_-CA) (S-NMe_3_Cl) (3)	435 ± 1	0.05 ± 0.01	54 ± 1	13

**Table 4:** Minimum inhibitory concentration (MIC) and minimum bactericidal concentration (MBC) against *S. aureus* and *E. coli* for (HS-G_1_NMe_3_Cl (**I**) and HS-PEG2K-CA (**1**)) AuNPs(S-PEG2K-CA) (**2**) and AuNPs((S-PEG2K-CA) (S-G_1_-NMe_3_Cl) (**3**)).

Compound	*S. aureus*		*E. coli*	
	MIC (mg/L)	MBC (mg/L)	MIC (mg/L)	MBC (mg/L)
I	4	8	16	16
1	> 1024	> 1024	> 1024	> 1024
2	> 1024	> 1024	> 1024	> 1024
3	256	512	> 1024	> 1024

## Data Availability

All data supporting the results are included within the article and in the Supporting Information.

## Supporting Information

Supporting information associated with this article includes:

Table S1. TEM and DLS characterization data of AuNPs.

Figure S1 Study of the antioxidant activities in a wide range of concentrations and times by DPPH assay.

Figure S2. Study of the antioxidant activities in a wide range of concentrations and times by FRAP assay.

Scheme S1. Reversible redox process of caffeic acid.

Figure S3. Electrochemical characterization A) CV of free CA (black) and free thiolate ligand (green) at the same concentration of CA moieties. Measurements were recorded on SPCE in contact with an aqueous 0.1M phosphate buffer solution (pH = 7.4) at a scan rate 50 mV·s^−1^.

Figure S4. Determination of the diffusion coefficient (A) and HET (B) for free CA molecules in aqueous 0.1M phosphate buffer solution (pH = 7.4). CA equivalent concentration was 50 μM in all the samples.

Figure S5. Plot of the cathodic and anodic peak potentials of the S-PEG-CA ligand anchored to an Au surface as a function of the scan rate.

Figure S6. CV for the RD process of the CA-PEG-S-Au SAM formed in a gold electrode at a modification time of 16 h, in KOH 0.1 M. The thin black line corresponds to the naked electrode. Sean rate: 0.02 V/s.
