# Supplementary material for: Enhancing Polyphenol Delivery and Efficacy Using Functionalized Gold Nanoparticles: Antioxidant and Antibacterial Properties
Source: Bioinorg Chem Appl. 2025 Jun 9;2025:3836765. doi: 10.1155/bca/3836765 (PMC12170072; doi:10.1155/bca/3836765)
Supplement: Supporting Information — Additional supporting information can be found online in the Supporting Information section. [file 3836765.f1.docx]

Manuscript submitted to ***Bioinorganic Chemistry and Applications***

**SUPPORTING MATERIAL**

**Enhancing Polyphenol Delivery and Efficacy Using Functionalized Gold Nanoparticles: Antioxidant and Antibacterial Properties.**

AuNPs(S-PEG-CA) (**2**) and AuNPs(S-PEG2K-CA)(S-G_1_-NMe_3_Cl) (**3**) capping ligands estimation.

The nanoparticle average diameter core obtained by TEM (**1.0 nm diameter**) was used to determine the gold atoms (N_Au_) in AuNPs **2 and** **3** core assuming spherical nanoparticles shape using the sphere volume formula,

$$V= \frac{4}{3}\pi r^{3}= \left( \frac{4}{3}\pi\right) \left( \frac{D}{2} \right)^{3}=0.52 {nm}^{3}$$

From the values of atomic weight and density of the gold atom, the number of atoms present in 1 nm^3^ was calculated.

Pm Au = 196.96 g/mol,

ρ Au = 19.3 g/cm^3^

N. Avogadro = 6.02x10 ^23^ atoms/mol

(19.3 g/cm3)/(196.96 g/mol) = (0.098 mol/cm^3^)(1 cm^3^/1 x 10 ^21^nm^3^)

= (9.799 x 10 ^-23^ mol/nm^3^)(6.022 x 10 ^23^ atoms/nm^3^) = 59 atoms/nm^3^

Thus, the number of Au atoms in a single AuNP,

$$N_{Au}=\left( 59 \frac{Au atoms}{{nm}^{3}} \right)\cdot0.52 {nm}^{3}=31 atoms Au/AuNP$$

The number of ligands present on the nanoparticle surface is calculated using the Au/ligand ratio, obtained from the thermogravimetric analysis: where the weight loss in the different range of temperatures that capping ligands degrade and their corresponding molecular weight allowed calculating the quantity of each capping ligand grafted to AuNPs.

For AuNPs(S-PEG-CA) (**2**): S-PEG-CA (PEG) (300-600 ºC), 85% and Au (residue), 15 %.

$$N_{\mathrm{PEG}}=\frac{0.85{g_{Dend}}/{g_{AuNps}}}{2132.56{g_{PEG}}/{{mol}_{PEG}}}=0.00384 \frac{{mol}_{PEG}}{g_{AuNps}}$$

$$N_{\mathrm{Au}}=\frac{0,15{g_{Au}}/{g_{AuNps}}}{196.96{g_{Au}}/{{mol}_{Au}}}=0.00761 \frac{{mol}_{Au}}{g_{AuNps}}$$

Then, Au/L molar ratios were obtained as follow:

$$\frac{Au}{S-PEG-CA}= \frac{0.0762 \frac{{mol}_{Au}}{g_{Au}}}{0.0384 \frac{{mol}_{PEG}}{g_{Au}}}=1.9844 \frac{{mol}_{Au}}{{mol}_{PEG}}$$

$$N_{L}= \frac{N_{Au}}{\left( \frac{Au}{L} \right)}$$

$$N_{PEG}= \frac{31}{1.9844}=15.42\cong15$$

And, considering the area of the AuNP core, the surface coverage (Γ) can be expressed as follows:

$$A_{AuNP}= 4 \pi r^{2}= \left( 4\pi\right) \left( \frac{1.0 nm}{2} \right)^{2}=3.14 {nm}^{2}=314 Å^{2}$$

$$\Gamma=\frac{314 Å^{2}}{16 molecules} \simeq21 Å^{2}/S-PEG2K-CA$$

For AuNPs(S-PEG-CA)(S-G_1_-NMe_3_Cl) (**3**): S-NMe_3_Cl (Dend) (100-300 ºC), 7 %; S-PEG-CA (PEG) (300-600 ºC), 66 % and Au (residue), 27 %.

$$N_{\mathrm{Dend}}=\frac{0.07{g_{Dend}}/{g_{AuNps}}}{496.77{g_{Dend}}/{{mol}_{Dend}}}=0.000141 \frac{{mol}_{Dend}}{g_{AuNps}}$$

$$N_{\mathrm{PEG}}=\frac{0.66{g_{Dend}}/{g_{AuNps}}}{2132.56{g_{PEG}}/{{mol}_{PEG}}}=0.000309 \frac{{mol}_{PEG}}{g_{AuNps}}$$

$$N_{\mathrm{Au}}=\frac{0,27{g_{Au}}/{g_{AuNps}}}{196.96{g_{Au}}/{{mol}_{Au}}}=0.001371 \frac{{mol}_{Au}}{g_{AuNps}}$$

Then, Au/L molar ratios were obtained as follow:

$$\frac{Au}{Dend}= \frac{0.001371 \frac{{mol}_{Au}}{g_{Au}}}{0.000141 \frac{{mol}_{Dend}}{g_{Au}}}=9.72 \frac{{mol}_{Au}}{{mol}_{Dend}}$$

$$\frac{Au}{S-PEG-CA}=4.20 \frac{{mol}_{Au}}{{mol}_{PEG}}$$

N_Au_ and corresponding Au/L molar ratios of each ligand made possible to calculate the number of different ligands (N_L_) grafted to the AuNPs surface:

$$N_{L}= \frac{N_{Au}}{\left( \frac{Au}{L} \right)}$$

$$N_{Dend}= \frac{31}{9.72}=3.18 \cong3$$

$$N_{PEG}= \frac{31}{4.20}=7.38 \cong7$$

And, considering the area of the AuNP core, the average surface coverage (Γ) could be estimated as:

$$\Gamma=\frac{314 Å^{2}}{10 molecules} \simeq31 Å^{2}/molecule$$

Estimation of ^a^ the ligands anchored to each AuNP and ^b^ total surface coverage based on the results obtained from TGA analysis (% of organic matter) and TEM (1.0 nm AuNP size)

| AuNP | molecule | % TGA | L/AuNP ^a^ | Γ (Å^2^/molecule) ^b^ |
| --- | --- | --- | --- | --- |
| 2 | -S-PEG2K-CA | 85 | 16 | 21 |
| 3 | -S-PEG2K-CA | 66 | 7 | 31 |
|  | -S-G_1_-NMe_3_Cl | 7 | 3 |  |

|  |  | **Intensity-based distributions** | | | **Volume-based distributions** | | |
| --- | --- | --- | --- | --- | --- | --- | --- |
| **AuNPs** | **D^a^** | **PDI^b^** | **D_x_^c^** | **CD_x_^d^** | **PDI^b^** | **D_x_^c^** | **CD_x_^d^** |
| **2** | 1 | 0.37 | 75.91±3.09 | 15.67 | 0.26 | 7,71±1,35 | 2,41 |
| **3** | 1 | 0.36 | 53.61±3.62 | 13.35 | 0.27 | 8,25±1,91 | 2,46 |

**Table S1**. ^a^Diameter average size (nm) obtained by TEM. Diameter obtained by DLS using intensity and volume-based distributions: ^b^Polydispersity Index (PDI) in DLS measurements. ^c^Hydrodynamic diameter (d.nm) obtained by DLS. ^d^Calculated diameter. ^e^Zeta Potential (mV).


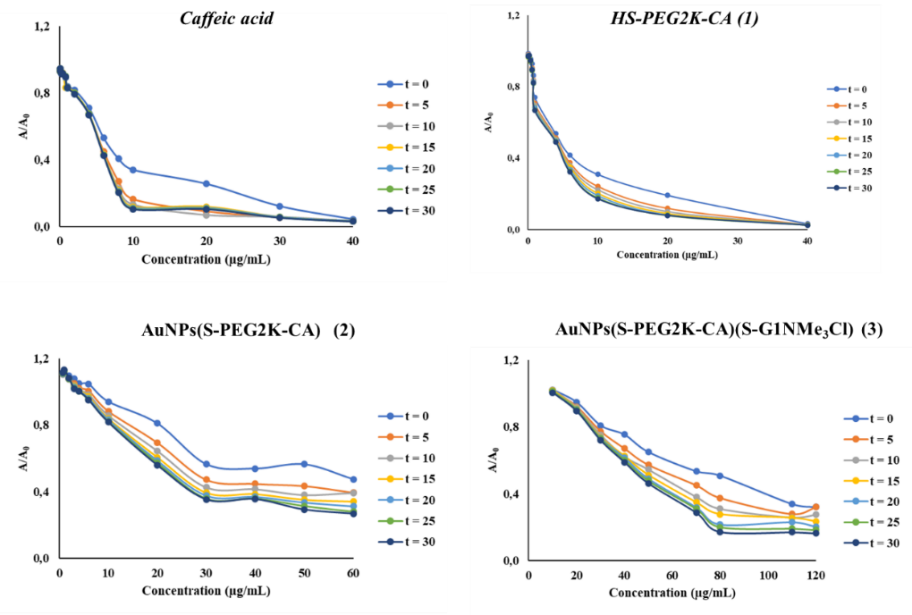


**Figure S1** Study of the antioxidant activities in a wide range of concentrations and times by DPPH assay


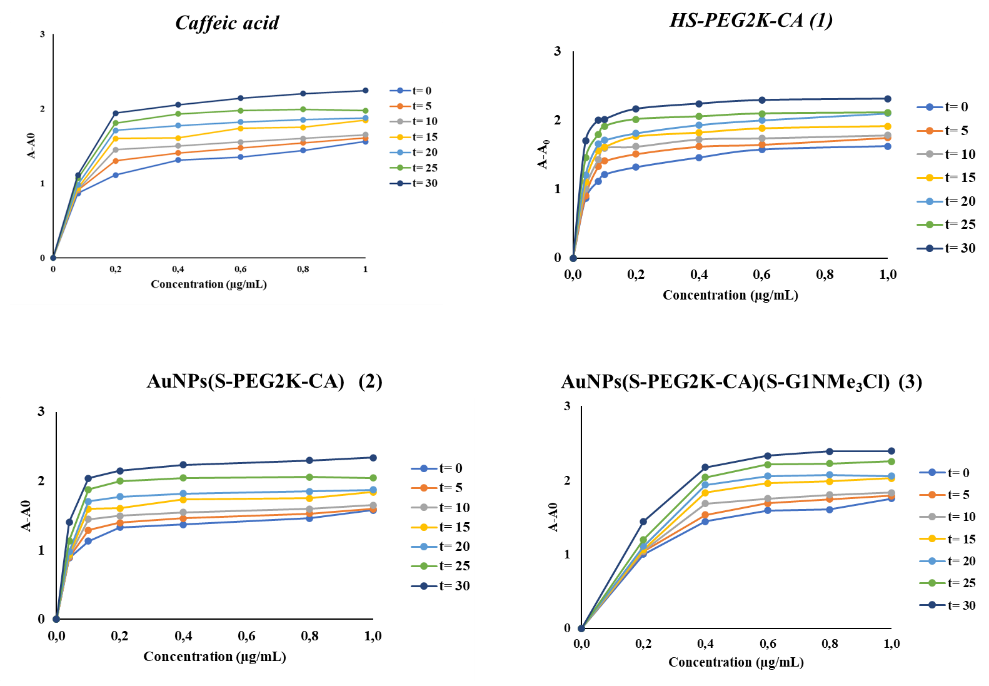


**Figure S2.** Study of the antioxidant activities in a wide range of concentrations and times by FRAP assay.


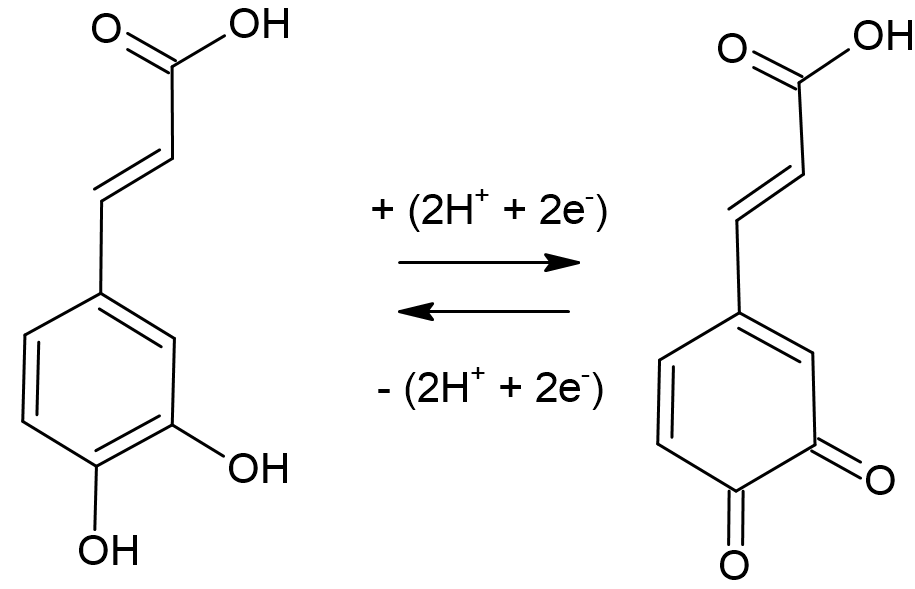


**Scheme S1**. Reversible redox process of caffeic acid.





**Figure S3**. A) CV of free CA (black) and free thiolate ligand (green) at the same concentration of CA moieties. B) CV of CA (black), AuNP **2** (blue) and AuNP **3** (red) at the same concentration of CA moieties. Measurements were recorded on SPCE in contact with an aqueous 0.1M phosphate buffer solution (pH=7.4) at a scan rate 50 mV·s^-1^

***Calculus of diffusion coefficient (D_0_) and HET (***$\text{k}^{\text{0}}$***)***

The diffusion coefficient (D_0_) is one of the most important characteristics of electroactive moieties in a molecule. Environmental variables greatly affect such parameters, including the electrolyte composition, temperature, and pressure. However, knowing its exact value seems to be essential for the understanding of electroactive species. The following equation, known as Randles-Sevcik equation, describes the effect of scan rate on the peak current i_p_ for a CV measurement at 25°C,

$$i_{p}=2.69 x {10}^{5}\cdot n^{3/2}\cdot A\cdot C\cdot D_{0}^{1/2}\cdot v^{1/2} (S1)$$

where i_p_ is the peak current of the redox reaction, n is the number of electrons involved, F is the Faraday’s constant in (C·mol^-1^), D_0_ is the diffusion coefficient (cm^2^·s^-1^), A is the area of the electrode (cm^2^) and C is the concentration of the redox molecule (mol·cm^-3^). The Randles-Sevick plot, that is, peak intensity versus square root of the scan rate, confirms that a diffusion control process is being produced.

As the scan rate increases, the electron transfer process tends to become more irreversible. In this situation, it is possible to apply the so-called Nicholson method to determine the heterogeneous electron transfer (or rate) constant, k^0^, which shows the reversibility of a process (cm·s^-1^). The method is based on a relation between the peak separation in the VCs and a function ($\Psi$) given by the equation:

$$\Psi=\frac{\gamma\cdot k^{0}}{\sqrt{\pi\cdot D\cdot a}} (S2)$$

where γ = Do/D_R_, a = nFv/RT and v is the scan rate. If γ = 1:

$$\Psi=\frac{k^{0}}{\sqrt{\pi\cdot D\cdot\frac{n\cdot F}{R\cdot T}}}\cdot\frac{1}{v^{\frac{1}{2}}} (S3)$$

So,

$$k^{0}=\Psi\cdot\left( \pi\cdot D_{o}\frac{nFv}{RT} \right)^{\frac{1}{2}} (S4)$$

The method can be applied in the range of peak separation between 57mV to 250mV, and the obtained results evidence the degree of reversibility of the process.





**Figure S4.** Determination of the diffusion coefficient (A) and HET (B) for free CA molecules in aqueous 0.1M phosphate buffer solution (pH=7.4). [CA] equivalent concentration was 50 μM in all the samples.

***Laviron’s model***

The Laviron method is frequently employed for determining the electron transfer rate constant, k_s_. According to this model, at high scan rates, the values of E_p_ vary linearly with log v (Figure S3) and, since these branches reach values of ΔE>200/n mV, the Eqn. S5-S7 can be used to obtain both α and k_s_ parameters.

$$E_{p}=E^{{^{\circ}}^{'}}+\frac{2.303RT}{\left( 1-\alpha\right)nF}\left( \frac{\log\left( 1-\alpha\right)nF}{RT-logk_{s}} \right)+\frac{2.303RT}{\left( 1-\alpha\right)nFlogv} (S5)$$

and from it, Eqn. (S5) is employed to obtain k_s_,

$$\log k_{s}=\alpha\log\left( 1-\alpha\right)+\left( 1-\alpha\right)log\alpha-log\frac{RT}{nFv}-\frac{\alpha\left( 1-\alpha\right)nF\eta}{2.303RT} (S6)$$

However, a simpler method of determining the k_s_ can be achieved by applying the overpotential boundary condition, E_p_ – E_1/2_ = η = 0, so Eqn. (3) is simplified to Eqn. (4),

$$k_{s}= \frac{\alpha nFv}{RT} (S7)$$




**Figure S5.** Plot of the cathodic and anodic peak potentials of the S-PEG-CA ligand anchored to an Au surface as a function of the scan rate.





**Figure S6.** CV for the RD process of the CA-PEG-S-Au SAM formed in a gold electrode at a modification time of 16 h, in KOH 0.1 M. The thin black line corresponds to the naked electrode. Sean rate: 0.02 V/s.

***REFERENCES***

1. Chiorcea-Paquim, A. M., Enache, T. A., Gil, E. D. & Oliveira-Brett, A. M. Natural phenolic antioxidants electrochemistry: Towards a new food science methodology. *Compr Rev Food Sci Food Saf* **19**, 1680–1726 (2020).  DOI: [10.1111/1541-4337.12566](https://doi.org/10.1111/1541-4337.12566)
2. Chevion, S., Roberts, M. A. & Chevion, M. The use of cyclic voltammetry for the evaluation of antioxidant capacity. *Free Radic Biol Med* **28**, 860–870 (2000). <https://doi.org/10.1016/S0891-5849(00)00178-7>
3. Astruc, D. Electron-transfer processes in dendrimers and their implication in biology, catalysis, sensing and nanotechnology. *Nat Chem* **4**, 255–267 (2012). doi:10.1038/nchem.1304
4. Lavagnini, I.; Antiochia, R.; Magno, F. An Extended Method for the Practical Evaluation of the Standard Rate Constant from Cyclic Voltammetric Data. *Electroanalysis* **2004**, *16*, 505-506.
5. [**https://doi.org/10.1002/elan.200302851**](https://doi.org/10.1002/elan.200302851)
6. Nicholson, R.S. Theory and Application of Cyclic Voltammetry for Measurement of Electrode Reaction Kinetics. *Analytical Chemistry* **1965**, *37*, 1351-1355.
7. Laviron, e. A multilayer model for the study of space distributed redox modified electrodes .1. Description and discussion of the model. *Journal of electroanalytical chemistry* **112**, 1–9 (1980).
8. Rohanifar, A., Devasurendra, A. M., Young, J. A. & Kirchhoff, J. R. Determination of L-DOPA at an optimized poly(caffeic acid) modified glassy carbon electrode. *ANALYTICAL METHODS* **8**, 7891–7897 (2016).
9. Salvarezza, R. C. & Carro, P. The electrochemical stability of thiols on gold surfaces. *Journal Of Electroanalytical Chemistry* **819**, 234–239 (2018). <https://doi.org/10.1016/j.jelechem.2017.10.046>
10. Chavez, M. *et al.* Electrochemical evaluation of the grafting density of self-assembled monolayers of polyethylene glycol of different chain lengths formed by the grafting to approach under conditions close to the cloud point. *Journal Of Electroanalytical Chemistry* **913**, (2022). <https://doi.org/10.1016/j.jelechem.2022.116294>
